# Exploration of the truncated cytosolic Hsp70 in plants - unveiling the diverse T1 lineage and the conserved T2 lineage

**DOI:** 10.3389/fpls.2023.1279540

**Published:** 2023-11-16

**Authors:** Yi-Jing Chen, Sou-Yu Cheng, Cheng-Han Liu, Wen-Chieh Tsai, Hsin-Hsin Wu, Ming-Der Huang

**Affiliations:** ^1^ Department of Biological Sciences, National Sun Yat-sen University, Kaohsiung, Taiwan; ^2^ Institute of Tropical Plant Sciences and Microbiology, National Cheng Kung University, Tainan, Taiwan

**Keywords:** Hsp70, EEVD motif, truncated Hsp70, Lauraceae, gene cluster, T1 lineage, T2 lineage

## Abstract

The 70-kDa heat shock proteins (Hsp70s) are chaperone proteins involved in protein folding processes. Truncated Hsp70 (Hsp70T) refers to the variant lacking a conserved C-terminal motif, which is crucial for co-chaperone interactions or protein retention. Despite their significance, the characteristics of Hsp70Ts in plants remain largely unexplored. In this study, we performed a comprehensive genome-wide analysis of 192 sequenced plant and green algae genomes to investigate the distribution and features of Hsp70Ts. Our findings unveil the widespread occurrence of Hsp70Ts across all four Hsp70 forms, including cytosolic, endoplasmic reticulum, mitochondrial, and chloroplast Hsp70s, with cytosolic Hsp70T being the most prevalent and abundant subtype. Cytosolic Hsp70T is characterized by two distinct lineages, referred to as T1 and T2. Among the investigated plant and green algae species, T1 genes were identified in approximately 60% of cases, showcasing a variable gene count ranging from one to several dozens. In contrast, T2 genes were prevalent across the majority of plant genomes, usually occurring in fewer than five gene copies per species. Sequence analysis highlights that the putative T1 proteins exhibit higher similarity to full-length cytosolic Hsp70s in comparison to T2 proteins. Intriguingly, the T2 lineage demonstrates a higher level of conservation within their protein sequences, whereas the T1 lineage presents a diverse range in the C-terminal and SBDα region, leading to categorization into four distinct subtypes. Furthermore, we have observed that T1-rich species characterized by the possession of 15 or more T1 genes exhibit an expansion of T1 genes into tandem gene clusters. The T1 gene clusters identified within the Laurales order display synteny with clusters found in a species of the Chloranthales order and another species within basal angiosperms, suggesting a conserved evolutionary relationship of T1 gene clusters among these plants. Additionally, T2 genes demonstrate distinct expression patterns in seeds and under heat stress, implying their potential roles in seed development and stress response.

## Introduction

Chaperone proteins are responsible for facilitating the folding of client proteins during protein translation, utilizing a range of diverse mechanisms (Beissinger and Buchner, 1998; Agashe and Hartl, 2000; Frydman, 2001; Tiwari et al., 2023). They also assist in recognition and delivery of misfolded proteins to subsequent degradation when proper folding is not achieved (Balchin et al., 2016; Fernández-Fernández et al., 2017). Heat shock proteins (Hsps) are a class of chaperone proteins that can be classified into different families based on their molecular mass, including Hsp40, Hsp70, Hsp90, and Hsp110 (Hendrick and Hartl, 1993). Specifically, Hsp40, Hsp70, and Hsp110 play crucial roles in facilitating protein folding starting from the initiation of the client protein synthesis. The process by which Hsp70 facilitates protein folding is known as the Hsp70 cycle (Liberek et al., 1991; Szabo et al., 1994; Dragovic et al., 2006). It begins with the binding of ATP to Hsp70, followed by the binding of the client protein. Subsequently, ATP is hydrolyzed into ADP with the assistance of the J-domain containing proteins (JDPs) Hsp40, enabling the client protein to initiate folding. Once the folding process is completed, the nucleotide exchange factor (NEF) Hsp110 assists in the release of ADP from Hsp70, leading to the dissociation of the folded protein from Hsp70. However, if proper folding cannot be achieved with the assistance of Hsp70, these unfolded proteins are directed towards the Hsp90 cycle for further folding or to the proteasome for degradation (Mayer and Bukau, 2005; Rosenzweig et al., 2019).

Hsp70s play pivotal roles in maintaining protein homeostasis, especially under heat stress conditions (Fernández-Fernández and Valpuesta, 2018). Under heat stress, Hsp70 dissociates from the Hsp70-HSF (heat-shock transcription factor) complex and engages with unfolded and aggregated proteins, thereby triggering the activation of separated monomeric HSF molecules. Following this, the activated HSF molecules assemble into an active trimeric complex and translocate to the nucleus, where it binds to the heat shock elements (HSE) located upstream of the Hsp70 promoter, initiating the expression of Hsp70 (Abravaya et al., 1992; Schlesinger and Ryan, 1993; Rabindran et al., 1994). In plants, overexpression of Hsp70 has been demonstrated to enhance their tolerance to heat stress, whereas the knockout mutants exhibit reduced thermotolerance (Sung and Guy, 2003; Su and Li, 2008). Beyond its role in heat stress response, Hsp70 is also involved in various critical aspects of plant development, immune responses, and defense against viruses (Kim and Oglesbee, 2012; Mine et al., 2012; Berka et al., 2022). In Arabidopsis, single *Hsp70* knockout mutants may not exhibit apparent phenotypes, but the severity of developmental abnormalities becomes evident in double and triple knockout mutant plants (Leng et al., 2017). Furthermore, it has been observed that the expression level of Hsp70 increases upon infection by pathogens and viruses (Wang et al., 2020; Zhang and Yu, 2022). Intriguingly, in specific instances, Hsp70s interact with viral polymerases, promoting enhanced viral replication, facilitating the formation of viral replication complexes, and contributing to the stability of complex proteins. As a result, *Hsp70* knockout mutants demonstrate increased resistance to virus infection (Hafrén et al., 2010; Gorovits and Czosnek, 2017; Wu et al., 2023). Moreover, members of the Hsp70 family cooperate with other chaperone proteins, such as Hsp90, SGT1 (suppressor of G-two allele of Skp1), and RAR1 (required for Mla12 resistance), to form a complex that regulates immune sensor proteins known as NLRs (nucleotide binding domain leucine-rich repeat containing proteins) upon pathogen infection (Shirasu and Schulze-Lefert, 2003; Noël et al., 2007; Kadota and Shirasu, 2012; Park and Seo, 2015). Despite these intricate interactions, individual Hsp70 gene knockout mutants do not exhibit any discernible difference in resistance against pathogens (Jungkunz et al., 2011; Hýsková et al., 2021; Berka et al., 2022).

The protein structure of Hsp70 consists of five main parts, arranged in sequential order from the N-terminus to the C-terminus: the nucleotide binding domain (NBD), a linker region, the substrate binding domain β-sheet (SBDβ), the substrate-binding domain α-helical lid (SBDα), and the C-terminal domain (CTD) (Flaherty et al., 1990; Bertelsen et al., 2009). The NBD serves as the binding domain for ATP/ADP, while the SBDβ functions as the site where the client protein binds. The SBDβ possesses an eight-stranded β sandwich structure and harbors a hydrophobic pocket that facilitates interaction with the hydrophobic regions of unfolded client proteins. Acting as a bridge between the NBD and SBDβ, the linker region contains a binding sequence for DNAJ, a Hsp40 (Jiang et al., 2007). The SBDα, known as the lid domain, comprises five α-helices (αA–αE). Upon substrate binding, the SBDα undergoes conformational changes, closing the hydrophobic pocket. After the protein folding process is complete, the lid domain opens again, enabling the release of the client proteins (Bertelsen et al., 2009; Zhang P. et al., 2014). The CTD of cytosolic Hsp70 encompasses a G-rich sequence, with the conserved Glu-Glu-Val-Asp (EEVD) motif positioned at the terminal end of the protein (Sharma and Masison, 2009). In yeast, this G-rich sequence has been proposed to serve as the binding site for the prion protein Ure2, inhibiting fibril formation (Zeytuni and Zarivach, 2012). On the other hand, the conserved EEVD motif is involved in binding to proteins containing the tetratricopeptide repeat (TRP) domain, such as the co-chaperones HOP and CHIP (Carrigan et al., 2006; Assimon et al., 2015). HOP, also known as Hsp70/Hsp90 organizing protein, acts as an adaptor protein that connects Hsp70 and Hsp90, facilitating the transfer of client proteins from Hsp70 to Hsp90 (Baindur-Hudson et al., 2015; Wang et al., 2022). CHIP (Carboxyl terminus of Hsp70-interacting protein), an E3 ubiquitin ligase, serves as a molecular link between Hsp70 and the E2 ubiquitin-conjugating enzyme, promoting the ubiquitination of misfolded proteins and facilitating their degradation (VanPelt and Page, 2017; Wu et al., 2021).

The core structure and sequence of Hsp70 proteins are highly conserved across diverse organisms. However, certain non-canonical Hsp70 variants have been discovered, displaying truncations or altered protein sequences, notably lacking the conserved EEVD motif at the C-terminus. The first instance of this truncated form was identified in humans and rats, termed *Stch*, encoding an Hsp70 like protein (HSP70L) (Otterson et al., 1994). Subsequently, in Arabidopsis, two of these non-canonical Hsp70s, *AtHspT1* and *AtHsp70T2*, were identified and predicted to be unexpressed pseudogenes or encode plastid proteins, termed truncated Hsp70s (Lin et al., 2001; Sung et al., 2001). Despite lacking portions of the C-terminal domain and its EEVD motif, these Hsp70 variants have proven to be important functional proteins in their own right. The study of Hsp70 homologs has been more extensively conducted in yeast, where a total of seven cytosolic Hsp70 proteins have been identified (Ingolia et al., 1982; Craig and Jacobsen, 1985; Hallstrom et al., 1998). Among these, four (Ssa1–4) harbor the conserved EEVD motif, while the remaining trio (Ssb1, Ssb2, and Ssz1) lacks it (Werner-Washburne et al., 1987; Tripathi et al., 2017). Specifically, both Ssb1 and Ssb2 lack the CTD, and Ssz1 harbors an atypical NBD and the SBDβ, lacking both the SBDα and CTD. These truncated Hsp70s function as co-translational chaperones (Zhang Y. et al., 2014). Earlier studies have illuminated the co-translational folding process orchestrated by them. Firstly, Ssz1 interacts with Zuotin (Zuo1), a J-domain (JD)-containing Hsp40 protein, to form the ribosome-associated complex (RAC) (Hundley et al., 2002; Chiabudini et al., 2012). The collaboration between RAC and Ssb1/2 establishes a unique triad at the ribosome, facilitating the binding and co-translational folding of nascent peptides in a sequential manner (Gumiero et al., 2016; Chen et al., 2022). Upon RAC loading onto the ribosome, the SBDβ of Zuo1 binds to a short nascent peptide emerging from the ribosome exit tunnel, while the His-Pro-Asp (HPD) sequence within the JD is shielded by SBDβ in Ssz1. The elongating peptide induces a conformational change in RAC, positioning the Ssz1 (SBDβ) to bind the nascent peptide, leading to Ssz1 mobilization and dissociation from Zuo1. This exposes JD and the HPD sequence of Zuo1. Subsequently, Ssb1/2-ATP recognizes JD in Zuo1, facilitating the recruitment of Ssb1/2 to the ribosome and the transfer of the nascent peptide from Ssz1 to Ssb1/2-ATP (SBDβ). Meanwhile, the HPD sequence of Zuo1 activates the ATPase activity of Ssb1/2-ATP, causing a closure of the lid (SBDα) of Ssb1/2 and disrupting the association of Ssb1/2 and Zuo1. After establishing a high substrate-binding affinity due to the conformational change triggered by ATP hydrolysis, Ssb1/2-ADP proceeds to facilitate the co-translational protein folding process (Tsai and Douglas, 1996; Chen et al., 2022). Defects in any of the components (Ssb1/2, Ssz1, and Zuo1) lead to similar *in vivo* phenotypes, including susceptibility to cold, protein synthesis inhibitors (paromomycin), salt, and a range of cations (Nelson et al., 1992; Yan et al., 1998; Gautschi et al., 2001; Kim and Craig, 2005). In humans, the Hsp70T homolog, termed Hsp70L1, fulfills a comparable function to Ssz1 by participating in RAC formation assisted by MPP11, a Zou1 homologous protein (Otto et al., 2005; Jaiswal et al., 2011). However, a comparable mechanism has not yet been reported in plants. In Arabidopsis, two Hsp70T sequences, namely *AtHsp70T1* and *AtHsp70T2*, have been identified (Lin et al., 2001). The expression of *AtHsp70T1* is generally low but increases under drought stress conditions, while *AtHsp70T2* exhibits expression in seeds and is upregulated in response to heat stress. Despite these observations, the precise functions of Hsp70Ts in plants remain largely unknown (Kiyosue et al., 1994; Rizhsky et al., 2004; Banti et al., 2008).

The comprehensive identification of genes across genomes provides valuable insights into their distribution, classification, and functional analysis. In this study, we aim to shed light on the function of cytosolic Hsp70T by investigating their distribution and protein features. By analyzing genomic databases from 192 green algae and plant species, we have identified cytosolic Hsp70T genes and classified them into two distinct lineages: the first lineage, marked by a greater diversity of the encoded protein sequences (T1), and the second lineage, characterized by less diverse protein sequences (T2). Further protein analysis has subdivided the T1 lineage into four distinct subtypes according to the features of their truncated CTDs. Some of these subtypes have undergone expansion and turned into tandem gene clusters within plant genomes. These gene clusters display syntenic relationships within their respective botanical families and unveil a potential evolutionary linkage between the Laurales order and basal angiosperms. Furthermore, our RNA-seq analysis revealed distinct expression patterns of the T2 members in seeds, providing insights into their regulation under conditions of heat stress.

## Materials and methods

### Genome-wide identification and classification of the Hsp70 family

Genomic and protein data of green algae and plants were downloaded from the GenBank of NCBI (www.ncbi.nlm.nih.gov), JGI (https://genome.jgi.doe.gov/portal/), Ensembl Plants (https://plants.ensembl.org/), PLAZA (https://bioinformatics.psb.ugent.be/plaza/), TAIR (https://www.arabidopsis.org/), FernBase (https://fernbase.org), Hornworts genome database in University of Zurich (https://www.hornworts.uzh.ch/en.html), and Bamboo genome database (http://www.genobank.org/bamboo) (**Supplementary Table 3**). To identify Hsp70 proteins, candidate sequences containing the Hsp70 motifs were selected using the HMMER program with a downloaded Hidden Markov Model (HMM) profile PF00012 from InterPro (www.ebi.ac.uk/interpro/). The E-value was set to 1e-20. The redundant protein sequences and isoforms were detected and removed using the TBLASTN program with genomic DNA sequences as the targets. Subsequently, a size selection criterion was applied, excluding sequences with erroneous annotations or other proteins also possessing the Hsp70 motifs, such as the Hsp110, based on a molecular weight range of 50–90 kDa. The protein molecular mass was computed using the Bio.SeqUtils package of Biopython (Cock et al., 2009). For the Hsp70 classification, the protein sequences were subjected to the BLASTP analysis against the reference sequences of Arabidopsis, including the sequences of cyHsp70, Bip, mtHsp70, and cpHsp70, using an E-value threshold of 1e-100. Sequences that could not be classified into any of the four types were excluded from further analysis.

To identify the conserved C-terminal motif, the last 20 amino acid residues from the C-terminus of the Hsp70 proteins from the same type were aligned using the MUltiple Sequence Comparison by Log-Expectation (MUSCLE) program (Edgar, 2004). The resulting alignment was then visualized using the online tool WebLogo (https://weblogo.berkeley.edu) and utilized for the identification of truncated Hsp70Ts. Hsp70 proteins lacking the conserved C-terminal motif were selected and classified as a distinct group known as truncated Hsp70 (Hsp70T) using a custom Python script.

### Motif finding and the classificaion of cyHsp70Ts

To determine the conserved motif patterns of full-length cyHsp70s, a comprehensive analysis was conducted on more than 1,000 protein sequences sourced from plants and green algae. Before initiating motif prediction, the protein sequences were segmented into discrete domains, including the nucleotide binding domain (NBD), linker, substrate binding domain β (SBDβ), substrate binding domain α (SBDα), and C-terminal domain (CTD), guided by the alignment outcomes. This segmentation was conducted to enhance the ease of subsequent analytical procedures. Motif discovery was performed using the MEME suite program, with a focus on identifying conserved sequences based on the lowest E-value and P-value. Notably, six conserved sequences were identified in the NBD, one in the linker, one in the SBDβ, four in the SBDα, and two in the CTD. These identified conserved sequences were subsequently utilized for motif searching of cyHsp70Ts using the Motif Alignment and Search Tool (MAST) within the MEME Suite, employing an E-value threshold of 1e-7. Following the classification, T1 proteins were further categorized into four distinct subtypes based on the variation or absence of the conserved sequences in the SBDα and CTD regions of the full-length cyHsp70. Furthermore, the protein sequences of both T1s and T2s were employed to identify conserved motifs specific to each lineage and subtype.

### Multiple sequence alignment and phylogenetic tree construction

To enable sequence comparison, the MUSCLE program was employed to perform multiple sequence alignment. The resulting alignment was subsequently visualized using the online tool ESPript 3 (https://espript.ibcp.fr). The phylogenetic tree was constructed using the Neighbor-join (NJ) method in the PHYLIP package, based on the multiple sequence alignment produced by the MAFFT program with default settings. Bootstrap values were generated using the resampling method with multiple datasets generated by the Seqboot program in the PHYLIP package (Retief, 2000), and the values were calculated using the Booster program (Lemoine et al., 2018). The final phylogenetic tree was visualized using the online iTOL program (https://itol.embl.de/).

### Protein similarity calculation

To assess the protein similarity between full-length and truncated cyHSP70s, we utilized the BLASTP program. The reference dataset comprised the 18 full-length cyHSP70s from 18 representing species, including *Arabidopsis thaliana*, *Populus trichocarpa*, *Oryza sativa*, *Phoenix dactylifera*, *Litsea cubeba*, *Cinnamomum micranthum*, *Persea americana*, *Nymphaea colorata*, *Amborella trichopoda*, *Picea abies*, *Thuja plicata*, *Ceratopteris richardii*, *Salvinia cucullata*, *Selaginella moellendorffii*, *Physcomitrella patens*, *Marchantia polymorpha*, *Klebsormidium nitens*, *Chlamydomonas reinhardtii*. We computed either the full-length sequences or partial sequences from the five major domains of the cyHSP70s against the 18 reference sequences to determine their average protein similarity.

### The syntenic gene analysis

To conduct syntenic analysis, ten genes located upstream and downstream of the T1 gene clusters were selected for homologous genes identification. The genome locations of their homologous genes were retrieved with the TBLASTN program. Synteny at a genomic position in this study is marked by the occurrence of three homologous genes within the neighboring set of ten genes. The syntenic clusters obtained were visually represented using Circos, a visualization tool generated through the R programming language Circlize (Krzywinski et al., 2009; Gu et al., 2014).

### RNA-seq database analysis

Raw data for the RNA-seq analysis were obtained from the Sequence Read Archive (SRA) of the National Center for Biotechnology Information (NCBI) website (https://www.ncbi.nlm.nih.gov/sra) and the reads were extracted using the SRA toolkit (https://hpc.nih.gov/apps/sratoolkit.html). The reads were then mapped to either the reference transcriptome or genomic sequence using the HISAT2 program (Kim et al., 2019). The resulting mapping files were sorted with SAMtools (Li et al., 2009), and RNA expression levels of target genes were estimated based on the mapped reads using StringTie with default settings (Pertea et al., 2015). To visualize transcript abundance levels, we employed the heatmap package in R language. **Supplementary Table 4** provides the accession numbers and other relevant information for the RNA-seq databases used in this study.

### Protein three-dimensional structure modeling

The 3D structures of both truncated and full-length cyHSP70s were predicted using the online program SWISS-MODEL (https://swissmodel.expasy.org/) (Waterhouse et al., 2018). Firstly, the protein sequences were aligned to identify the template structure with the highest GMEQ (global model quality estimation) score for homology modeling. Subsequently, the ProMod3 program was employed to generate the initial structures, and the structure with the highest QMEAN (Qualitative Model Energy Analysis) score was selected as the final model (Studer et al., 2021). The protein structures were visualized using PyMOL Molecular Graphics System, Version 2.5.1 (Schrödinger, 2015).

### *Ka*/*Ks* ratio calculation

The *Ka*/*Ks* ratio between two protein sequences was determined using the program Ka/Ks calculator (Wang et al., 2010). Before the calculation, two protein sequences were aligned using the MAFFT program. The aligned protein sequence was then converted to a codon alignment of the corresponding coding sequence (CDS) using a Perl script termed pal2nal.pl (Suyama et al., 2006). The *Ka*/*Ks* ratio was calculated based on the codon alignment result using the Ka/Ks calculator.

## Results

### *Hsp70Ts* are ubiquitously present in plants and green algae, with *cyHsp70Ts* being particularly abundant in plants

Plant cells exhibit four distinct forms of Hsp70 proteins: cytosolic Hsp70 (cyHsp70), endoplasmic reticulum (ER) Hsp70 (Bip, Binding immunoglobulin protein), mitochondrial Hsp70 (mtHsp70), and chloroplast Hsp70 (cpHsp70). To investigate the presence of truncated Hsp70 proteins in plants and green algae, we conducted a genome-wide search of Hsp70 genes in over 192 plant and green algae genomes. Our analysis includes 20 green algae (18 chlorophytes and two charophytes), six non-vascular plants (two hornworts, two liverworts, and two mosses), and 166 vascular plants. The vascular plants comprise one lycophyte, two ferns, eight gymnosperms, three basal angiosperms, one Chloranthales, eight Magnoliids, 38 monocots and 105 eudicots (**Supplementary Table 1**). In the initial phase, candidate genes encoding protein sequences containing the Hsp70 domains were identified. Following this, the molecular mass of these proteins was confined to a range of 50 to 90 kDa, ensuring the omission of inaccurately annotated sequences and other proteins featuring the Hsp70 domain, like Hsp110, which lie beyond this mass range. The classification of the four Hsp70 forms involved a comprehensive analysis using the BLASTP program and phylogenetic tree assessment. Given the conserved C-terminal motifs found within each Hsp70 form in eukaryotic cells, that is, EEVD for cyHsp70, HDEL for Bip, EEVKK for mtHsp70, and TDSK for cpHsp70, these conserved motifs were employed to discern the presence of truncated Hsp70 proteins (Hsp70Ts). Through an extensive search for genes encoding Hsp70 proteins devoid of the conserved C-terminal motifs, Hsp70Ts were successfully identified across all four Hsp70 forms. Detailed elucidation of the identification and classification of these Hsp70Ts can be found in the materials and methods section.

To determine the conservation of the C-terminal motif in both plants and green algae, a thorough comparative analysis of the conserved C-terminal sequences across the four Hsp70 forms was conducted within the investigated green algae and plant species (**Table 1**). The C-terminal sequences of cyHsp70s and Bips in ER exhibited distinctive conserved patterns shared across plant and green algae species. In contrast, the C-terminal motif of cpHsp70s displayed subtle variations between plant and green algae species. Additionally, the C-terminal sequence of mtHsp70 demonstrated slightly lower conservation in green algae compared to plants (**Table 1**; **Supplementary Figure 1**). Concretely, the conserved C-terminal sequences of cyHsp70s and Bips across plant and green algae species are GPKIEEVD and HDEL, respectively. For mtHsp70s, the C-terminal sequences are characterized by enriched Glu (E) and Lys (K) residues, exhibiting a consistent arrangement of EAEYEEVKK in plants. Notably, cpHsp70s demonstrated an Asp (D)-rich nature, with sequences DVIDADFTDSK/N and DVIDAEFTDKK corresponding to plants and green algae, respectively. Upon comparing these conserved sequences within full-length Hsp70s, our investigation shifted towards Hsp70Ts that lack these conserved motifs. Regarding the genes encoding the four forms of Hsp70T in plant and green algae species, approximately 45%, 15%, 10%, and 15% of the investigated green algae species were found to possess three or fewer genes for cyHsp70Ts, BipTs, mtHsp70Ts, and cpHsp70Ts, respectively (**Table 1**). In contrast, in plant species, approximately 98%, 40%, 23%, and 19% harbor cyHsp70Ts, BipTs, mtHsp70Ts, and cpHsp70Ts, respectively. Notably, the gene counts for plant BipTs, mtHsp70Ts, and cpHsp70Ts slightly surpass those in green algae. Noteworthy variation is observed in the gene count for cyHsp70Ts, which is significantly higher in plants, ranging from one to 58, with an average of 7.3 genes per species. Interestingly, the cyHsp70T gene is absent only in *Cuscuta australis* and *Kalanchoe fedtschenkoi* (**Supplementary Table 1**). These findings underscore the ubiquity and greater prevalence of cyHsp70Ts in plants compared to other truncated Hsp70 forms. Therefore, further examination is required.

**Table 1:** The gene number, occurrence rate, and the protein sequence of conserved C-terminal motif of full-length and truncated Hsp70s genes in a plant or green algae species.

	plant Hsp70s				algal Hsp70s			
	gene no. in a species		occurrence	C-terminal motif	gene no. in a species		occurrence	C-terminal motif
form	range	average			range	average		
cyHsp70s								
full-length	1–16	6.5	100%	GPKIEEVD	1–2	1.2	85%	GPKIEEVD
truncated	1–58	7.3	98%	–	1–3	1.3	45%	–
Bips								
full-length	1–15	3.4	100%	HDEL	1–3	1.2	80%	HDEL
Truncated	1–6	1.8	40%	–	1	1.0	15%	–
mtHsp70s								
full-length	1–9	2.7	100%	EAEYEEVKK	1–2	1.1	75%	E/K rich
Truncated	1–4	1.6	23%	–	1	1.0	10%	–
cpHsp70s								
full-length	1–8	2.5	100%	DVIDADFTDSK/N	1–3	1.2	90%	DVIDAEFTDKK
truncated	1–3	1.3	19%	–	1	1.0	15%	–

The compilation is based on the data extracted from the genomic sequences of 172 plant and 20 green algae species. The table provides the average number and range of full-length and truncated Hsp70 genes within each respective Hsp70 form for each species. The table presents the average number and range of genes encoding both full-length and truncated Hsp70s within each respective Hsp70 form for every species. Additionally, it displays the occurrence ratios of these genes among the investigated plant and green algae species. Instances where a conserved C-terminal motif sequence is absent are indicated by ‘-’.

### Among the two cyHsp70T groups, the T1 lineage exhibits higher sequence similarity with the full-length cyHsp70s compared to the T2 lineage

**Figure 1:**
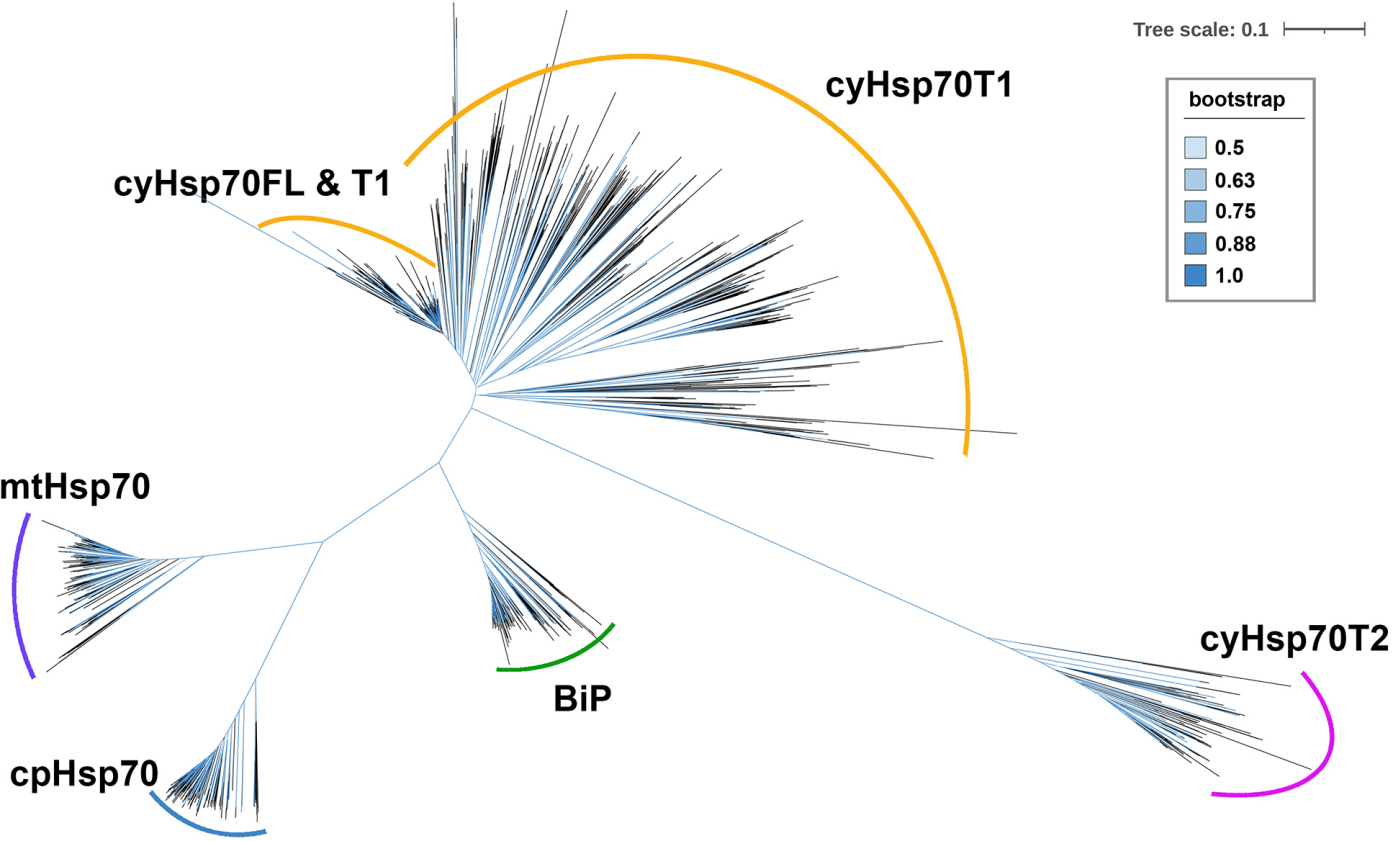
An unrooted phylogenetic tree of truncated cytosolic Hsp70s (cyHsp70Ts). A phylogenetic tree was constructed using protein sequences of cyHsp70Ts from all investigated species, along with sequences of four Hsp70 forms (cyHsp70s, Bips, mtHsp70s, and cpHsp70s) obtained from 18 representative plant and green algae species. The sequences were aligned using the MAFFT program, and an unrooted inference tree was generated using the neighbor-joining (NJ) method supported with 1,000 bootstrap resampling. Branches with bootstrap values exceeding 0.5 are shown in blue. The scale bar represents the unit of amino acid substitution per site. The cyHsp70FL, cyHsp70T1 and cyHsp70T2 represent the sequences of full-length cyHsp70s, the T1 lineage of truncated cyHsp70s, and T2 lineage of truncated cyHsp70, respectively.

**Figure 2:**
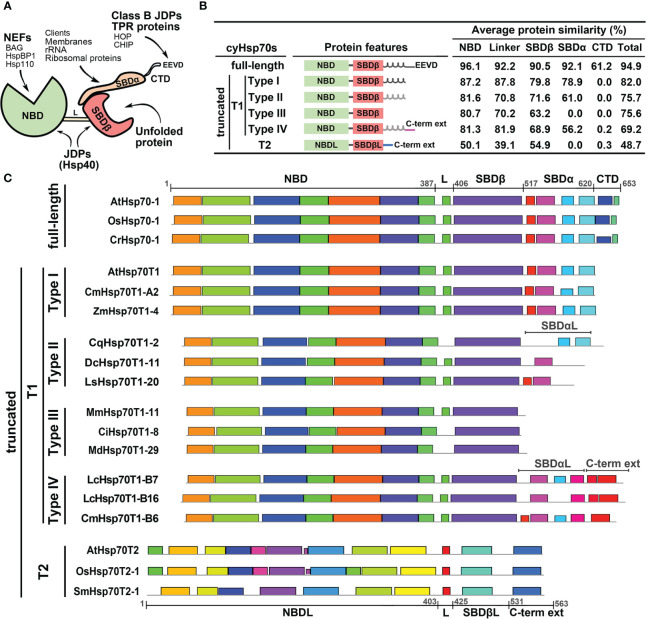
Structure and protein features of full-length and truncated cyHsp70s. **(A)** Schematic diagram of the full-length cyHsp70 protein structure. Arrows indicate potential interactions with specific domains or motifs of co-chaperones. **(B)** Sequence features and average protein similarities, expressed as percentages, between the two truncated lineages (T1 and T2) and full-length cyHsp70s. The analysis includes 1,049 full-length cyHsp70 protein sequences, 859 T1 sequences (comprising 127 Type I, 566 Type II, 118 Type III, and 48 Type IV), and 219 T2 sequences, with reference to 18 full-length cyHsp70 sequences. General sequence features of each cyHsp70 type are illustrated alongside the analysis. The black and gray spiral spring lines situated adjacent to the SBDβ domain within the protein features column delineate SBDα and the atypical SBDα domain (SBDαL), respectively. **(C)** The patterns of conserved sequences within each cyHsp70s type. The conserved sequence blocks of the representative cyHsp70 sequences were identified using the MEME suite. The cyHsp70s representative sequences of each type were collected from *Arabidopsis thaliana*, *Daucus carota*, *Lactuca sativa*, *Mikania micrantha*, *Cichorium intybus*, *Malus domestica golden*, *Oryza sativa*, *Zostera marina*, *Chenopodium quinoa*, *Litsea cubeba*, *Cinnamomum micranthum*, *Selaginella moellendorffii*, and *Chlamydomonas reinhardtii*. L, SBDβL, SBDαL and C-term ext indicate the linker, atypical SBDβ, atypical SBDα, and the C-terminal extension regions, respectively.

To further examine the cyHsp70Ts, we performed a phylogenetic analysis using all the putative protein sequences of cyHsp70s identified in aforementioned 192 species along with the sequences of full-length Hsp70 of all four Hsp70 forms obtained from 18 representative plant and green algae species, such as *Arabidopsis thaliana*, *Physcomitrella patens* and *Chlamydomonas reinhardtii*, etc. The full list is provided in the section of Material and Methods. In the phylogenetic tree, sequences of cyHsp70Ts form two distinct clades, namely T1 and T2 lineages (**Figure 1**). Notably, within the T1 lineage, a remarkable observation is that 6.9% of the T1 members are integrated within the same cluster as the full-length cyHsp70 sequences. These T1 members within the integrated cluster exhibit fewer deletions and sequence variations, indicating a closer resemblance to the full-length cyHsp70s. The remaining 93.1% of T1 sequences are positioned in separate clusters near the full-length cyHsp70s, implying a higher degree of diversity among these T1 sequences. On the other hand, the phylogenetic tree revealed that the T2 lineage exhibits distant relation to both the T1 lineage and full-length cyHsp70s, suggesting lower sequence similarity shared by both T1 members and full-length cyHsp70s. In Arabidopsis, two cyHsp70T genes were identified, namely *AtHsp70T1* and *AtHsp70T2*. The phylogenetic tree indicated that AtHsp70T1 belongs to the T1 group, while AtHsp70T2 is classified into the T2 lineage. Furthermore, we also conducted sequence similarity analysis on the protein sequences of T1s, T2s, and full-length cyHsp70s. Considering that the sequence similarities among the full-length cyHsp70s across species are generally above 85% (data not shown), we utilized a protein sequence of the full-length cyHsp70s from each of the 18 representative plant and green algae species as the reference for sequence similarity computation. The examination of sequence similarity among five major regions of these cyHsp70 groups were also examined (**Figures 2A, B**). The results showed an average of 94.9% similarity among the full-length cyHsp70s, with similarity exceeding 90% in the NBD, linker, SBDβ, and SBDα regions (**Figure 2B**). However, the average sequence similarity in the CTD is approximately 61.2%, indicating a diverse nature of this region. Moreover, the average protein similarity between the members of T1s and full-length cyHsp70 exceeds 69%, whereas the average protein sequence similarity between the members of T2 and full-length cyHsp70 is only 48.7% (**Figure 2B**). This significant difference in sequence similarity further emphasizes the distinctness of the T2 group from both the T1 and full-length cyHsp70s.

### T1 lineage exhibits significant sequence variation and encompasses four subtypes, whereas T2 lineage are conserved

The phylogenetic tree analysis revealed a significant degree of diversity within the T1 lineage, necessitating the classification of these sequences. We observed that the sequences of T1 lineage not only lack the C-terminal EEVD motif but also exhibit different levels of deletions in the SBDα or CTD regions. Methods like phylogenetic trees or motif analyses face challenges in classifying these sequences due to their considerable variability in both the SBDα and CTD these regions. Thus, the classification of the T1 members was based on the extent of variation or truncation of the SBDα or CTD regions (**Figure 2C**). Through a comparison of their protein similarity and the patterns of the conserved sequence blocks with full-length cyHsp70s, we divided the T1 sequences into four subtypes, denoted as Type I to IV. The Type I sequences were characterized by a deletion in the CTD region; Type II sequences exhibited a deleted CTD along with an atypical SBDα; Type III sequences displayed deletions in both the CTD and SBDα regions; and Type IV sequences possessed an atypical SBDα followed by an extended C-terminal domain, which differs from the full-length one but remains conserved among species. Additionally, the protein three-dimensional (3D) structure modeling of the T1 lineage proteins revealed a resemblance to the full-length cyHsp70, particularly in the NBD and SBDβ regions. Notably, significant differences among these subtypes (Type I to IV) were observed mainly in the CTD and SBDα regions (**Supplementary Figures 2**, **3**).

Compared to the T1 lineage, the protein sequence similarity between T2 and full-length cyHsp70s is relatively low, averaging below 55% across the linker and other regions (**Figure 2B**). Notably, T2 sequences possess an atypical NBD (NBDL) connected by a linker to an atypical SBDβ (SBDβL), along with a distinctive C-terminal extension sequence, while lacking the SBDα region. While none of the conserved sequence blocks found in full-length cyHsp70 were identified in the T2 sequences (**Figure 2C**), sequence analysis and 3D protein structure modeling indicate that T2 proteins still exhibit similarities to full-length cytosolic Hsp70 in terms of the arrangement of secondary structures, including the presence of three major nucleotide-binding interaction sites in the NBDL and a β-sandwich structure in SBDβL (**Supplementary Figures 2**, **4**). However, protein similarities among T2 members are less diverse, with an average similarity of 84.8% to each other (data not shown). Several conserved sequence blocks were observed in the four structural domains illustrated in **Figure 2C**: NBDL, the Linker (L), SBDβL, and the C-terminal extension sequence. Furthermore, the protein sequence of T2 contains a Serine (S) and Threonine (T)-rich region of approximately 20 amino acid residues in the N-terminal region upstream of NBDL, as well as a C-terminal extension with α-helix structures (**Supplementary Figures 2**, **4**).

### The T1 lineage is widespread among green algae and has diversified into a large family in plants, whereas the T2 lineage is exclusively found in plants with a limited gene number

**Figure 3:**
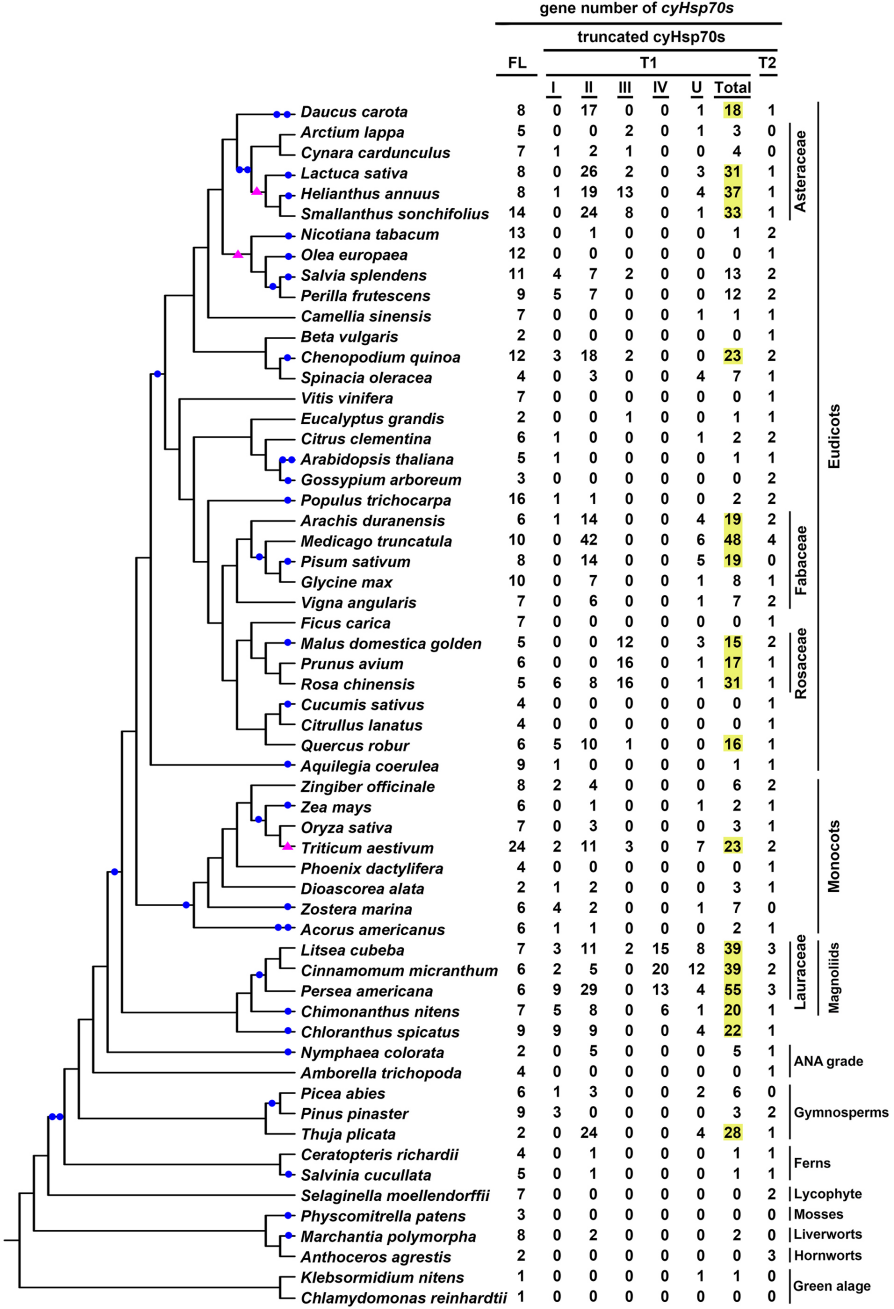
Gene numbers of full-length and different truncated cyHsp70 types in plant and green algae. The distribution of full-length cyHsp70 genes (FL) and cyHsp70T genes (T1, with I–IV subtypes, and an unidentified U group; and T2) in plants and algae is presented in the phylogenetic analysis of the investigated species. T1 gene numbers exceeding 15 are highlighted in yellow. Filled blue circles and pink triangles on the branches of the phylogenetic tree indicate whole-genome duplication and triplication events, respectively.

The cyHsp70T genes identified in green algae and plant species were classified into two lineages: T1 (comprising four subtypes, Type I-VI) and T2. Upon examination of the gene distribution, it was observed that T1 genes are present in both green algae and plants, while T2 genes are exclusive to plants and absent in green algae (**Figure 3**; **Supplementary Table 1**). The gene number of the T1 lineage in a species ranges from one to 55, with an average of 7.3, while the gene number of the T2 lineage in a species ranges from one to four, with an average of 1.3. Among the 20 green algae species studied, 45% of them possess T1 genes, with the number ranging from one to three. All of these algal sequences belong to Type II T1 lineage (**Figure 3**; **Supplementary Table 1**). In plants, T1 genes were detected in 65.7% of the investigated species, 81.9% of which have less than ten T1 genes. Overall, 14.6% of the T1 sequences identified in plants are Type I, 56.7% are Type II, 11.8% are Type III, 5.2% are Type IV, while 11.8% remain unclassified (**Supplementary Table 1**). Notably, Type III sequences are predominantly present in species of Rosaceae and Asteraceae, while Type IV sequences are exclusive to species of Laurales. Moreover, among the investigated plant species, there are 26 considered T1-rich plants, possessing 15 or more T1 genes in their genomes (**Supplementary Table 2**). These T1-rich plants comprise two gymnosperms, two monocots and 22 dicots, belonging to 11 families, Notably, four of these families have three or more T1-rich species, originating from different orders, including Asteraceae, Fabaceae, Rosaceae, and Lauraceae (**Figure 3**). In contrast to T1 lineage genes, the T2 genes are exclusively found in plants, occurring in up to 92.7% of the studied plants. Among the six non-vascular plants investigated, only the two hornworts, *Anthoceros agrestis* and *Anthoceros punctatus*, contain the T2 lineage genes. The number of T2 genes in these species is all less than five, and 82% of the investigated plant species have one to two the gene in their genomes.

### The genes of the T1 lineage that expanded into tandem gene clusters in T1-rich plants have a close evolutionary relationship within their respective plant family

Gene duplication events can occur through various mechanisms such as whole genome duplication, tandem duplication, retroduplication, and others (Lallemand et al., 2020). To investigate the potential correlation between the T1 gene duplications in T1-rich plants and whole genome duplication events, we compiled a collection of documented genome duplication instances in plants and green algae (Clark and Donoghue, 2018; Ren et al., 2018; Qiao et al., 2022). Concurrently, we cataloged the counts of each cyHsp70 type and incorporated this data into our phylogenetic analysis of the investigated species (**Figure 3**). We found that the increased T1 gene numbers do not exhibit substantial correlation with the occurrence of genome duplication or triplication events, except for the case of wheat (*Triticum aestivum*). This observation gains additional significance from the fact that neither the number of full-length cyHsp70 genes nor that of T2 genes exhibits a parallel escalation in these T1-rich plants, implying an autonomous expansion of T1 genes independent from whole genome duplication events. In contrast, both *Triticum turgidum* (a tetraploid) and *T. aestivum* (a hexaploid) exhibit a Hsp70 gene count approximately two to three times higher compared to *Aegilops tauschii* (a diploid) (**Supplementary Table 1**), across nearly all four Hsp70 forms. To further investigate the occurrence of duplication events of T1 genes in T1-rich plants, we considered their gene locations. Our findings revealed that T1 genes are predominantly found as gene clusters within these T1-rich plants, except for *T. turgidum* and *Pinus taeda* (**Supplementary Table 2**). Moreover, the number of T1 gene clusters in a species ranges from one to seven, with each cluster containing three to 24 T1 genes (**Supplementary Table 2**). Notably, six species, namely *Salvia hispanica*, *Chenopodium quinoa*, *Medicago truncatula*, *Rosa chinensis*, *Cinnamomum micranthum*, and *Litsea cubeba*, harbor a T1 gene cluster with more than 10 genes. Remarkably, the largest T1 gene cluster was observed in *L. cubeba*, forming a supercluster consisting of 12, seven, and five genes in the three subclusters.

**Figure 4:**
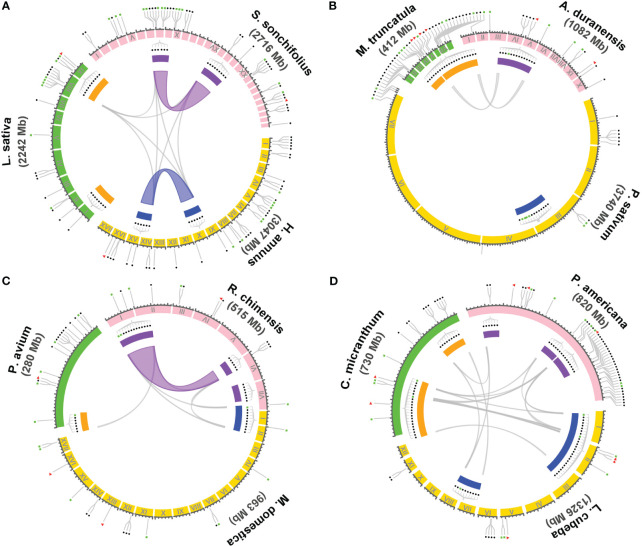
Syntenic analysis of the gene cluster of T1 lineage within and between species. The syntenic relationship of the genome regions containing T1 gene clusters in the genomes of species from four dicot families, including Asteraceae, Fabaceae, Rosaceae, and Lauraceae, were performed using Circos plot. Three representative species were chosen from each family for the analysis, including **(A)** Asteraceae: *Smallanthus sonchifolius*, *Helianthus annuus*, and *Lactuca sativa*. **(B)** Fabaceae: *Arachis duranensis*, *Pisum sativum*, and *Medicago truncatula*. **(C)** Rosaceae: *Rosa chinensis*, *Malus domestica golden*, and *Prunus avium*. **(D)** Lauraceae: *Persea americana*, *Litsea cubeba*, and *Cinnamomum micranthum*. T1 gene clusters containing six or more T1 genes are denoted with filled rectangles in different colors enclosed by the synteny circle. Clusters with a close evolutionary relationship in a particular species are highlighted with the same color either in orange, purple, and blue by the gene cluster. Colored and gray curves represent the syntenic regions and genes of the T1 gene clusters among and across species, respectively. The gene positions of full-length *cyHsp70s*, unclustered T1 or the cluster with less than six T1 genes, and T2 genes are indicated respectively with filled red triangles, blue circles, and green rectangles outside of the synteny circle.

Within the 14 families encompassing the T1-rich plants, four families, namely Asteraceae, Fabaceae, Rosoideae, and Lauraceae, exhibit three or more genera with T1 gene clusters. To investigate the evolutionary relationship of these T1 gene clusters, we conducted a syntenic gene analysis on the clusters containing more than five T1 genes. The shared intra- and inter-species synteny among the T1 gene clusters in these families is illustrated in **Figure 4**. The analysis of intra-species synteny revealed that the T1 gene clusters are conserved in two Asteraceae species (*Smallanthus sonchifolius* and *Helianthus annuus*) and one Rosoideae species (*Rosa chinensis*). Additionally, the inter-species synteny analysis demonstrated that the synteny of T1 gene clusters is conserved across species belonging to the same family, except for the case where there is only one species (*Pisum sativum*) in the Fabaceae family. Among the species harboring multiple T1 gene clusters, it is noteworthy that the three Lauraceae species exhibit highly conserved syntenic regions within all their T1 gene clusters across species.

### The T1 gene clusters in Lauraceae species exhibit conservation with those found in Chloranthales and basal angiosperms

**Figure 5:**
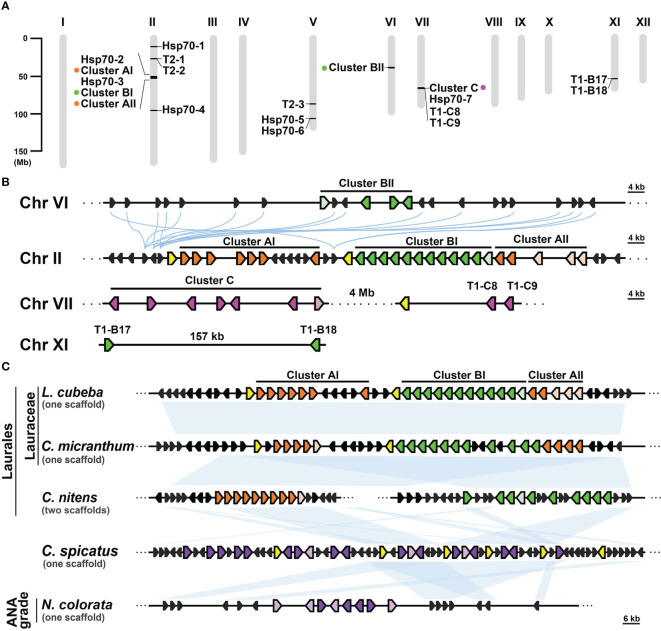
Syntenic analysis of T1 gene clusters in Laurales, Chloranthales, and basal angiosperms. **(A)** The chromosomal distribution of cyHsp70 genes and gene clusters in the *Litsea cubeba* genome (LcHsp70s). **(B)** Syntenic gene analysis and the relative positions and orientations of *T1* and their neighboring genes. Blue curved lines represent the gene synteny relationships across chromosomes. **(C)** Syntenic gene analysis focusing on the supercluster of T1 genes at Chromosome II of *L. cubeba*. Syntenic analysis of the *T1* supercluster was performed with three Laurales species (*L. cubeba*, *Cinnamomum micrathum*, and *Chimonanthus nitens*), one Chloranthales species (*Chloranthus spicatus*), and one basal angiosperm species (*Nymphaea colorata*). Syntenic regions are shown as blue blocks. T1 genes of Cluster A, B, and C are represented by filled orange, green, and magenta pentagons, respectively. Purple pentagons denote the T1 genes at the chromosomes of *C. spicatus* and *N. colorata*, while the pentagons filled with light orange, light green, light magenta, and light purple indicate the T1 genes lacking the N-terminal regions. Yellow and black pentagons respectively represent the full-length cyHsp70s and their neighboring genes. Scale bars on the side indicate the length of the analyzed scaffolds in kilobases.

Gene clusters can emerge through gene duplication events and often share a common ancestral origin between species (Makarova et al., 2005; Bharadwaj et al., 2021). In the case of the T1 gene clusters observed in Lauraceae species, their remarkable conservation of syntenic regions suggests a shared ancestry among these clusters and their close relatives. To investigate this further, we conducted a syntenic gene analysis using *L. cubeba* as a reference due to its well-annotated genome at the chromosome level. In *L. cubeba*, we identified a total of seven full-length cyHsp70, 39 T1, and three T2 genes. The putative proteins encoded by these T1 genes exhibit diversity, but the members within the same T1 gene cluster show sequence conservation. According to the phylogenetic analysis focused on the T1 sequences of *L. cubeba* and two other Lauraceae species, *Cinnamomum micranthum* and *Persea americana*, the T1 sequences could be categorized into three groups: Group A, Group B, and Group C (**Supplementary Figure 5**). Members of Group A belong to Type II T1, while sequences of Group B and Group C belong to Type IV T1. The C-terminal extension of the Group B proteins is more conserved than that of the Group C proteins, featuring a highly conserved leucine-rich region in an α-helix structure (**Supplementary Figure 6**). Thus, the T1 gene clusters of Lauraceae are also divided into three types, namely Cluster A, Cluster B, and Cluster C, based on the presence of Group A, B, and C genes. The 39 T1 genes in *L. cubeba* are distributed across four chromosomes (chromosomes II, VI, VII, and XI), with 35 of them organized into three gene clusters on chromosomes II, VI, and VII (**Figures 5A, B**). Among the three clusters, there is one supercluster located on chromosome II containing 24 T1 genes, further divided into three subclusters, namely Clusters AI, BI, and AII, according to the aforementioned groups to which the genes belong. The syntenic gene analysis revealed that the supercluster on chromosome II shows synteny with Cluster BII on chromosome VI but not with Cluster C on chromosome VII (**Figure 5B**). In the genomic database, we identified only one close relative of Lauraceae, *Chimonanthus nitens*, belonging to the Laurales order. *C. nitens* possesses two T1 gene clusters, namely cluster A and cluster B, located on separate scaffolds. Both clusters display synteny with the T1 supercluster found in *L. cubeba* and *C. micranthum*. Additionally, we observed that both *Chloranthus spicatus* (a Chloranthales species) and *Nymphaea colorata* (a basal angiosperm) also possess one T1 gene cluster, exhibiting synteny with the T1 gene cluster found in Laurales (**Figure 5C**). Notably, the T1 gene cluster of *C. spicatus* consists of seven full-length cyHsp70 genes. However, we did not observe the presence of the T1 gene cluster in the other four Magnoliids (*Liriodendron chinense*, *Magnolia officinalis*, *Aristolochia fimbriata* and *Aristolochia contorta*) and in the two basal angiosperms (*Nymphaea thermarum* and *Amborella trichopoda*).

### The transcripts of T1 genes are hard to detect and T2 genes are specially-expressed in the seed and induced by heat stress

**Figure 6:**
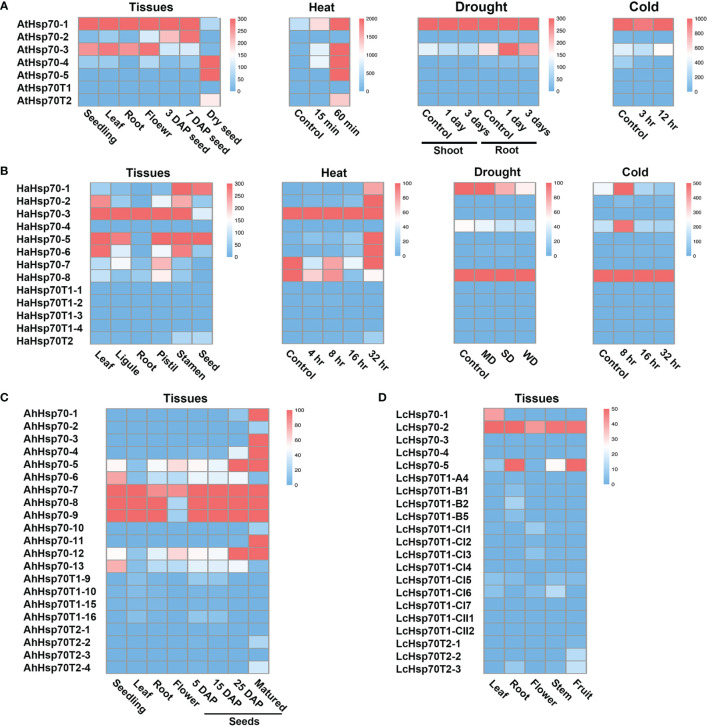
Transcript levels of cyHsp70Ts in various tissues, developmental stages and under different stress treatments. Transcript levels of cyHsp70s in several conditions of **(A)** *A. thaliana*, **(B)** *Helianthus annuus*, **(C)** *Arachis hypogaea*, and **(D)** *Litsea cubeba* were determined based on RNA-seq databases. The transcript levels are represented as FPKM (Fragments Per Kilobase of transcript per Million mapped reads) values and visualized as a heat map. ND, MD, and WD represent mild drought stress (MD), severe drought stress (SD), and rehydration to normal water supply after 3 days of severe drought (WD) in *H. annuus*. For detailed information on these RNA-seq databases, please refer to **Supplementary Table 4**.

In Arabidopsis, T1 genes generally show low expression levels, while T2 genes are specifically expressed in seeds and induced by heat stress (Lin et al., 2001; Rizhsky et al., 2004). To explore the expression patterns of cyHsp70Ts in representative plants, we examined their transcript levels utilizing RNA-seq data sourced from the NCBI database. The obtained data were subsequently visualized through heatmaps. The generated heatmaps distinctly illustrate that transcripts of T1 genes were scarcely detectable across various Arabidopsis tissues, as well as under conditions of heat, cold, and drought stress (**Figure 6A**). Conversely, AtHsp70T2 demonstrates distinct expression patterns, being specifically expressed in dry mature seeds, with no significant expression observed in developing seeds or other tissues. Additionally, AtHsp70T2 transcripts are detected under heat stress conditions, while remaining absent during cold or drought stresses. Transcript levels analysis of *cyHsp70Ts* in *Helianthus annuus*, *Arachis hypogaea* and *L. cubeba* showed extremely low expression of T1 genes in these species (**Figures 6B–D**). In contrast, *T2 gene* transcripts were detected in seeds and fruits of *H. annuus*, *A. hypogaea* and *L. cubeba*. Furthermore, *HaHsp70T2* transcripts were observed under 32 hours of heat stress but not under cold and drought stresses (**Figure 6B**).

## Discussion

The sequence and structural architecture of Hsp70 exhibit a high degree of conservation across diverse species, with a conserved C-terminal motif found in all four Hsp70 forms, cytosolic, ER, mitochondrial, and chloroplast. This conserved motif plays a pivotal role in facilitating protein interactions and intracellular targeting (Guy and Li, 1998; Smock et al., 2011). Given this context, it is plausible that truncated Hsp70 (Hsp70T) characterized by the absence of this conserved C-terminal motif, may serve distinct functional roles in cellular processes. In this study, we conducted a comprehensive analysis of genes encoding Hsp70Ts in both plants and green algae. Our investigation unveiled the distribution ratios of Hsp70Ts across all four Hsp70 forms, highlighting an abundance of the truncated cytosolic variants (cyHsp70Ts), which stand out as the most prevalent among the Hsp70Ts. These cyHsp70Ts were categorized into two lineages: T1, found in both green algae and plants, and T2, exclusive to plant species. Notably, T1 exhibits a pronounced similarity to the full-length cyHsp70, implying a potential evolutionary interconnection between T1 and the full-length cyHsp70. This similarity suggests that the emergence of both full-length and truncated forms could have potentially occurred through evolutionary processes in either direction. However, the emergence of T2 lineage appears to be a more recent event compared to T1. As a result, it remains challenging to definitively conclude the precise evolutionary relationship between T2 and T1, or between T2 and full-length Hsp70. The protein sequence of T2 displays lower similarity to that of full-length cyHsp70 than T1, and its conserved sequence patterns differ from both full-length cyHsp70 and T1. Furthermore, the gene of the T2 lineage lacks the intron typically present in both full-length cyHsp70 and T1, specifically at the codons coding for the conserved sequence Asp-Ala-Lys-Arg (DAKR) (data not shown, Lin et al., 2001). Moreover, a synteny analysis of the most primitive T2 sequences and other cyHsp70s, including full-length and T1 variants, in two hornwort species, *A. agrestis* and *A. agrestis*, did not reveal evident synteny (data not shown).

The primary function of cyHsp70 is to serve as chaperon protein that facilitates proper protein folding, especially when confronted with elevated temperature stress (Subjeck et al., 1982; Li et al., 1992). The higher gene counts of both full-length and truncated cyHsp70s in plant species, compared to green algae, suggests a potential adaptation of plants to the terrestrial environment characterized by more extreme temperature condition. Our observation also suggested a rapid gene expansion of the T1 lineage in plants hosting numerous T1 genes, referred to as T1-rich plants, leading to the formation of T1 gene clusters. Intriguingly, these T1-rich plants belong to 14 distinct plant families that are not closely related from an evolutionary standpoint, suggesting an independent origin of the T1 gene clusters within the species of these diverse plant families. Considering that the putative protein sequences encoded by T1 genes show a diversity of sequences and can be classified into four subtypes, this variability among T1 members not only reinforces the concept of independent expansion of the T1 gene cluster into a large gene family in the T1-rich species from different botanical families but also suggests a potential differentiation in functions for the T1 lineage. On the other hand, T2 sequences are more conserved, suggesting a potentially distinct role for T2 in plants.

The analysis of syntenic genes has revealed an intriguing evolutionary pattern: T1 gene clusters within species of the same botanical family exhibit a high degree of synteny, implying a close evolutionary relationship among these T1 gene clusters. This phenomenon is particularly pronounced within the context of the Lauraceae family, a constituent of the Laurales order situated within the magnoliids clad of core angiosperms. Among the members of the Lauraceae family, which includes species *L. cubeba*, *C. micranthum*, and *P. americana*, the T1 gene clusters composed of genes from Group A, B, and C demonstrate a conserved synteny across species. This shared synteny strongly hints at a common origin from an ancestral cluster, underscoring the evolutionary interconnectedness of these T1 gene clusters. Notably, the T1 supercluster exclusively identified within the Lauraceae family also displays synteny with clusters A and B of T1 genes present in a more distantly related species *C. nitens* (a member of the Laurales order), and with T1gene clusters of *C. spicatus* (belonging to Chloranthales) and *N. colorata* (a representative of Nymphaeales in basal angiosperms). These observations suggest a possibility that the ancestral T1 gene cluster may have arisen in the common ancestor of Laurales, Chloranthales and basal angiosperms. Subsequently, it could have undergone evolution, leading to the emergence of clusters A, and B within the Laurales order and further diversification into clusters A, B, and C within the Lauraceae family. Importantly, while the cluster A and cluster B in *C. nitens* genome are located on separate scaffolds, their counterparts within Lauraceae species exhibit adjacency. This suggests a hypothesis that T1 gene expansion could have been facilitated through chromosomal crossover events, potentially contributing to the amplification of gene clusters marked by synteny within the Lauraceae family. These findings collectively illuminate the dynamic evolutionary processes that have contributed to the emergence and diversification of T1 gene clusters across the plant species.

Hsp70s display a notable preservation in both their protein sequence and structural configuration, rendering any mutations within the sequence capable of perturbing or modifying their inherent functional characteristics (Baxter and Craig, 1998; Davis et al., 1999; Ran et al., 2004). The T1 lineage of cyHsp70T has been categorized into four distinct subtypes (Type I–IV) based on observed variations or deletions within the substrate binding domain α (SBDα) and the C-terminal domain (CTD). The CTD region features a conserved EEVD sequence at its C-terminus, which holds vital importance for interactions with co-chaperones like HOP and CHIP. The absence of the EEVD motif in T1 sequences suggests a deficiency in the capacity to transfer client proteins to Hsp90 or to the ubiquitin-proteasome pathway (Mayer and Bukau, 2005; Smock et al., 2011). The SBDα, characterized by constituted by a structure composed of secondary α-helices, functions as a lid that facilitates the rapid binding and dissociation of the client proteins. Furthermore, it engages in interactions with ribosomes and other proteins, exerting influence over the process of protein folding (Gumiero et al., 2016; Lee et al., 2021). In contrast, the missing or presence of variations in the SBDα region results in the impairment or reduction of the protein’s capacity to function as a foldase for proper protein folding. Instead, this altered version maintains its role as a holdase, engaging in the binding and stabilization of unfolded or misfolded proteins, thereby preventing their aggregation until they can undergo appropriate refolding or degradation processes (Mayer et al., 2000; Slepenkov and Witt, 2002). The Type I T1 protein sequence bears a deletion within the CTD region, while still preserving the ability to facilitate protein folding. However, it lacks the competence to transfer client proteins to Hsp90 or the proteasome. Type II T1, on the other hand, possesses a variable or partially truncated SBDα, suggesting its retained capacity for protein folding, albeit potentially with reduced efficiency compared to full-length cyHsp70s. Conversely, Type III T1 represents the lidless form of cyHsp70, signifying its deficiency in foldase capability, while still serving as a holdase. Moreover, the sequence of Type IV T1 sequence comprises an atypical SBDα (SBDαL) domain and a C-terminal extension, implying the potential for foldase activity, coupled with the possibility of interactions with other proteins. Of particular note, the Type IV sequences are exclusively identified within the Laurales order, indicating distinctive roles for these proteins specifically within plant species belonging to this botanical order. Within the T1 lineage of the Lauraceae family, the Group B genes, which belongs to Type IV T1, bear a conserved leucine-rich α-helix in their C-terminal extension. It is recognized that leucine-rich domains in LRR (leucine-rich repeat) proteins, or other proteins, frequently mediate protein-protein interactions. Hence, we speculate that the leucine-rich α-helix within Group B T1 proteins might also participate in protein-protein interactions. However, due to their low expression levels, drawing definitive conclusions about their roles within the Laurales order remains a challenge.

The T2 lineage exhibits a significant reduction in protein similarity to the full-length cyHsp70 in comparison to the truncated T1 lineage. However, it still maintains a similar secondary structure and possesses catalytic sites with identical functions situated in the NBD and SBDβ, akin to the full-length cyHsp70 and T1 variants. This preservation underscores that T2 maintains its functions in nucleotide and substrate binding. However, the T2 sequence is devoid of the SBDα, while exhibiting a C-terminal extension. This configuration suggests a holdase activity, accompanied by the acquired ability to interact with other proteins. RNA-seq analysis unveiled that T2 is exclusively expressed in dry seeds and during a 60 minutes of heat treatment, suggesting its involvement in seed development processes and heat-responsive reactions. T2 genes have been detected in almost all vascular plants, whereas in the six non-vascular plant species investigated, T2 genes were exclusively identified in hornworts. These observations suggest a potential origin of T2 genes in the shared ancestor of vascular and non-vascular plants, followed by a subsequent expansion and prevalence among vascular plants. Additionally, the *Ka*/*Ks* ratios, which indicate the ratio of non-synonymous to synonymous substitution rates, were calculated for T2 sequences from 18 reprehensive plants. The resulting values ranged from 0.03 to 0.74, with an average of 0.12. These ratios suggest that these genes are under the influence of purifying selection pressure in plants (data not shown).

Hsp70 serves as a central player in maintaining protein homeostasis by assisting in the protein folding process (Fernández-Fernández and Valpuesta, 2018). However, in the context of cytosolic truncated Hsp70 (cyHsp70T), characterized by a truncated CTD and an atypical or truncated SBDα, there might be a potential reduction in its efficiency in protein folding. This prompts speculation that cyHsp70T might uphold protein homeostasis through alternative mechanisms. Although the exact function of cyHsp70T in plants remains elusive, insights from studies in yeast and mammalian cells suggest their involvement in co-translational protein folding (Gautschi et al., 2001; Hundley et al., 2005; Otto et al., 2005). As mentioned earlier, in yeast, counterparts of plant truncated cytosolic Hsp70s, Ssb1/2 (lacking CTD) and Ssz1 (bearing atypical NBD and SBDβ while lacking both the SBDα and CTD), collaborate with Zou1. Specifically, the ribosome-associated complex (RAC) formed by Ssz1 and Zou1 recruits and facilitates Ssb1/2 binding to nascent peptides near the ribosome exit tunnel. (Hundley et al., 2002; Huang et al., 2005; Zhang et al., 2017; Lee et al., 2021). Similarly, human Hsp70L achieves protein folding through the RAC complex with MPP11, a Zou1 homologous protein (Otto et al., 2005). In plants, the existence of the RAC complex has not been documented, primarily because Zou1 homologous genes are generally absent. In this study, we conducted a search for Zou1 homologs in the investigated databases using the HMMER program and found one gene encoding Zou1-like proteins respectively in *Spinacia oleracea* (XP_021856403.1) and *Cichorium endivia* (KAI3476403.1). Although both of these Zou1-like proteins were identified to have the DNAJ (PF00226) and ribosome-associated complex head domains (PF16717) according to Pfam analysis, they were annotated as unknown proteins, suggesting their limited resemblance to Zou1. Nevertheless, it is plausible that cyHsp70T in plants may still function as a co-translational chaperone by interacting with other proteins that have ribosome interaction domains. Additionally, yeast Ssb1/2 and Ssz1 exhibit limited ATPase activity compared to their full-length counterparts (Huang et al., 2005; Conz et al., 2007). Notably, Ssz1, with an atypical NBD, binds nucleotides but lacks ATPase activity. Therefore, investigating the ATPase activity of cyHSP70Ts, especially the T2 members possessing an atypical NBD, would hold promise. Although the ATPase activity of these cyHSP70Ts has not been determined, our observation in the T1-rich plant *L. cubeba* revealed no corresponding increase in the count of nucleotide exchange factor (NEF) genes, such as *Hsp40s* and *Hsp110s*. This suggests that cyHsp70T might be intricately involved in protein homeostasis pathways that are less reliant on high nucleotide hydrolysis rates.

It is worth mentioning that cyHsp70T has been reported to function as a pivotal translocation motor during the posttranslational import of cytoplasmic proteins into the endoplasmic reticulum (ER), mitochondria, and chloroplasts (De Los Rios et al., 2006; Craig, 2018; Shan, 2023). Yeast Ssa1 and Ssb1 interact with the TPR motif of Sec72 to facilitate protein translocation to the ER (Tripathi et al., 2017; Graham et al., 2019). Ssb1, lacking the EEVD motif, binds to the ribosome-nascent chain complex and interacts with Sec72 through the NBD. Furthermore, Hsp70 aids in protein translocation into mitochondria and chloroplasts by forming a guide complex with 14-3-3 proteins in plants (May and Soll, 2000). However, no evidence of interaction between plant cyHsp70T and 14-3-3 proteins have been documented. Interestingly, the subcellular localization of Arabidopsis T2 (AtHsp70T2) is diverse, spanning not only the cytosol, but also chloroplasts, and peroxisomes, but excluding the nucleus (Lin et al., 2001; Chowdhary et al., 2012). This hints at a plausible role for AtHsp70T2 as a translocation motor in the cytosol. However, more comprehensive experimental analysis is necessary to unveil the precise functions of cyHsp70T.

## Conclusion

The cytosolic Hsp70T variants (cyHsp70T) exhibit a notable conservation and wide distribution across plant species. Our extensive analysis elucidates the distribution patterns and distinctive attributes of cyHsp70T within plants, emphasizing the predominance of cytosolic Hsp70T and delineating distinct lineages within this subgroup. These findings further contribute to our understanding of the evolutionary dynamics and potential functional significance of cyHsp70T in various plant species.

## Data availability statement

The original contributions presented in the study are included in the article/**Supplementary Material**, further inquiries can be directed to the corresponding author/s.

## Author contributions

YC: Data curation, Formal Analysis, Investigation, Methodology, Software, Validation, Writing – original draft, Writing – review & editing, Visualization. SC: Writing – original draft, Writing – review & editing, Validation, Visualization. CL: Writing – review & editing, Validation, Visualization. WT: Validation, Writing – review & editing. HW: Writing – review & editing, Visualization. MH: Conceptualization, Formal Analysis, Funding acquisition, Investigation, Methodology, Project administration, Software, Supervision, Validation, Writing – original draft, Writing – review & editing, Resources, Visualization.
