# Supplementary material for: Exploration of the truncated cytosolic Hsp70 in plants - unveiling the diverse T1 lineage and the conserved T2 lineage
Source: Front Plant Sci. 2023 Nov 16;14:1279540. doi: 10.3389/fpls.2023.1279540 (PMC10687569; doi:10.3389/fpls.2023.1279540)
Supplement: Supplementary file 1 [file DataSheet_1.pdf]

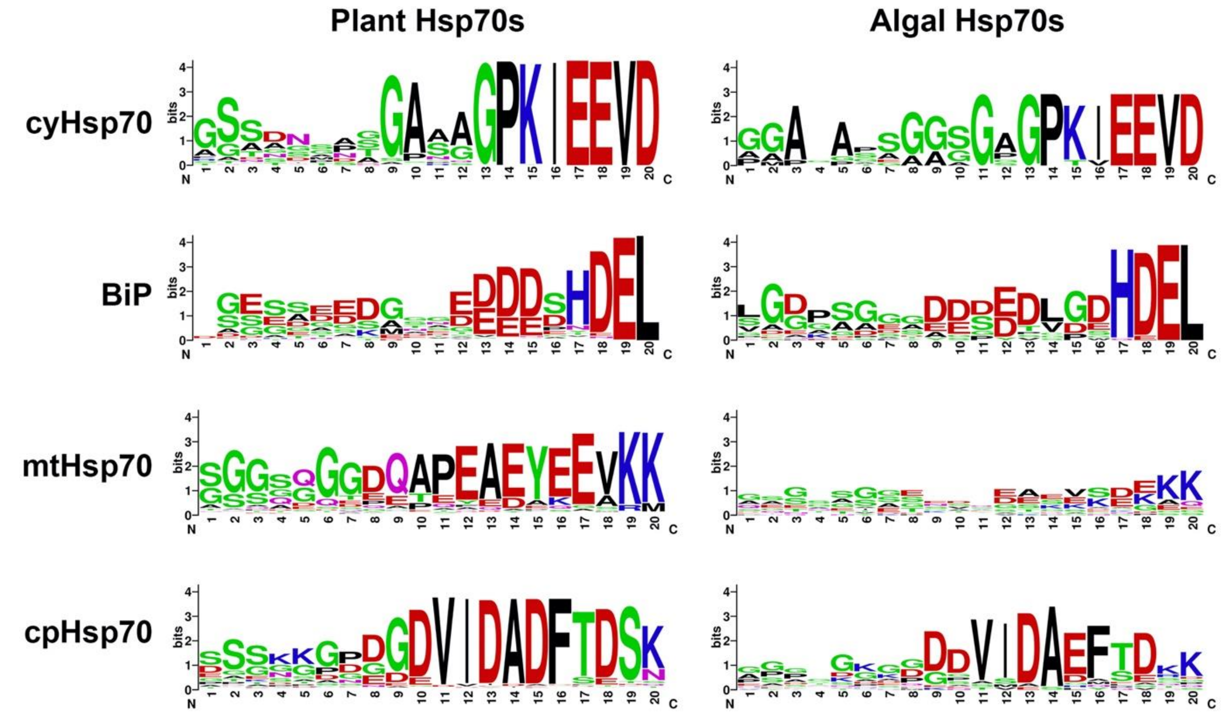

Supplementary Figure 1. Conserved C-terminal motifs of Hsp70s. The conserved C-terminal sequence of Hsp70s were determined by aligning the final 20 amino acids residues from the C-terminus using the MUSCLE program. The resulting sequence alignment was visualized as residue frequency at each position, employing the online tool WebLogo (https://weblogo.berkeley.edu/logo.cgi). This analysis utilized the protein sequences of Hsp70 obtained from 172 plants and 20 green algae.

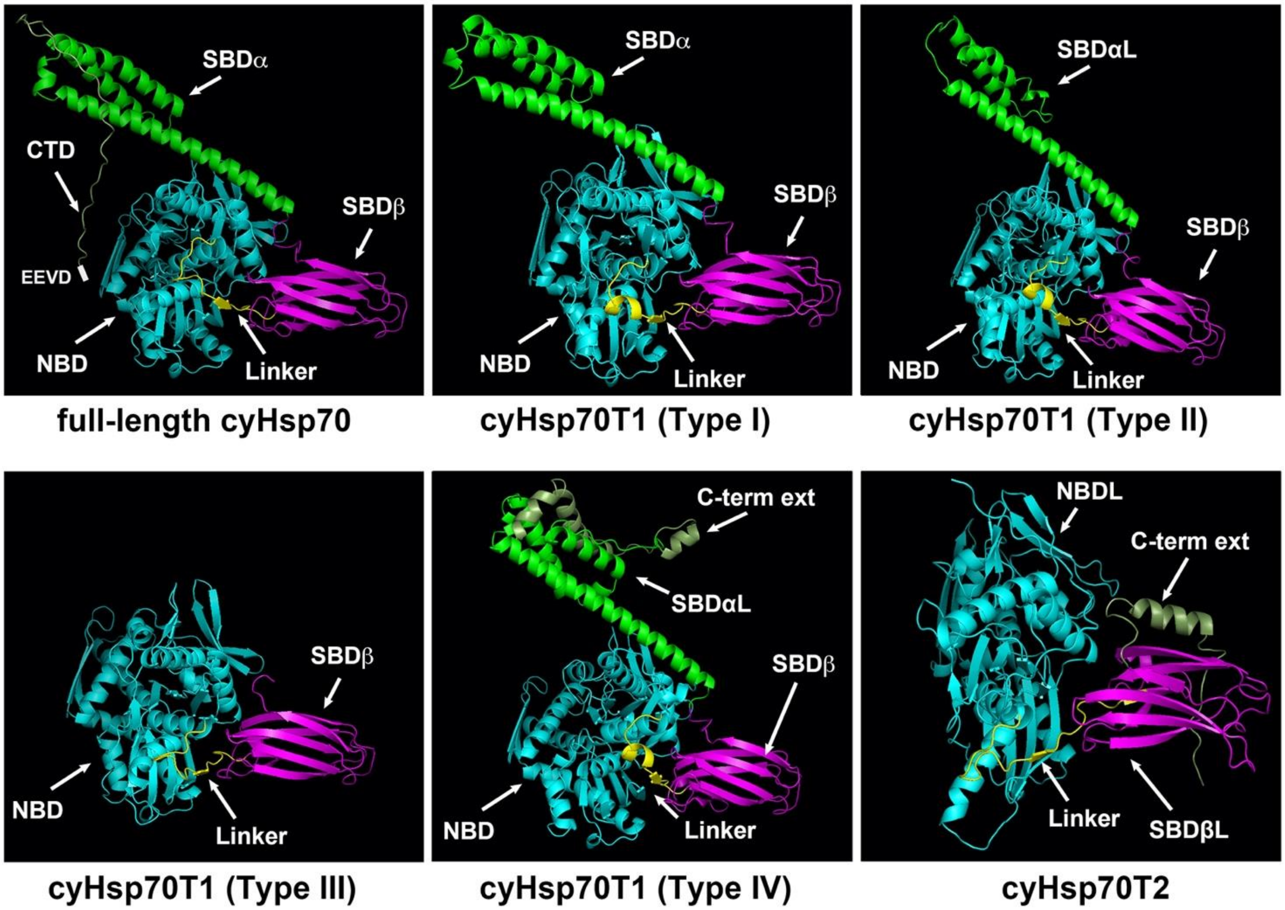

Supplementary Figure 2. Protein 3D modeling of all cyHSP70 types. The three-dimensional structures of both full-length and truncated cyHSP70 proteins were predicted using the online tool SWISS-MODEL (https://swissmodel.expasy.org/). The respective protein IDs from the genomic database and the corresponding SMTL IDs for the modeled proteins in SWISS-MODEL for each cyHSp70 type are provided as follows: full-length cyHsp70: AT5G02500 (*Arabidopsis thaliana*) / A0A0R4J626 (*Glycine max*); cyHsp70T1 (Type I): AT1G56410 (*A. thaliana*) / A0A3Q7Y9C5 (*Cicer arietinum*); cyHsp70T1 (Type II): PaHsp70T1-B14 (*Persea americana*) / A0A1S3TT80 (*Vigna radiata* var radiata); cyHsp70T1 (Type III): MD14G0022900 (*Medicago truncatula*) / A0A6P5R6Z9 (*Prunus avium*); cyHsp70T1 (Type IV): LiHsp70T1-B14 (*Litsea cubeba*) / A0A3S4NG45 (*Cinnamomum micranthum* f kanehirae); cyHsp70T2: AT2G32120 (*A. thaliana*) / I1JAU2 (*G. max*). Distinct color codes represent the five major regions of Hsp70 (NBD, linker, SBDβ, SBDα and CTD). The term "C-term ext" designates the C-terminal extension. The atypical NBD, SBDβ and SBDα regions are labeled as NBDL, SBDβL and SBDαL, respectively. The conserved C-terminal motif "EEVD" of full-length cyHsp70 is highlighted in white.

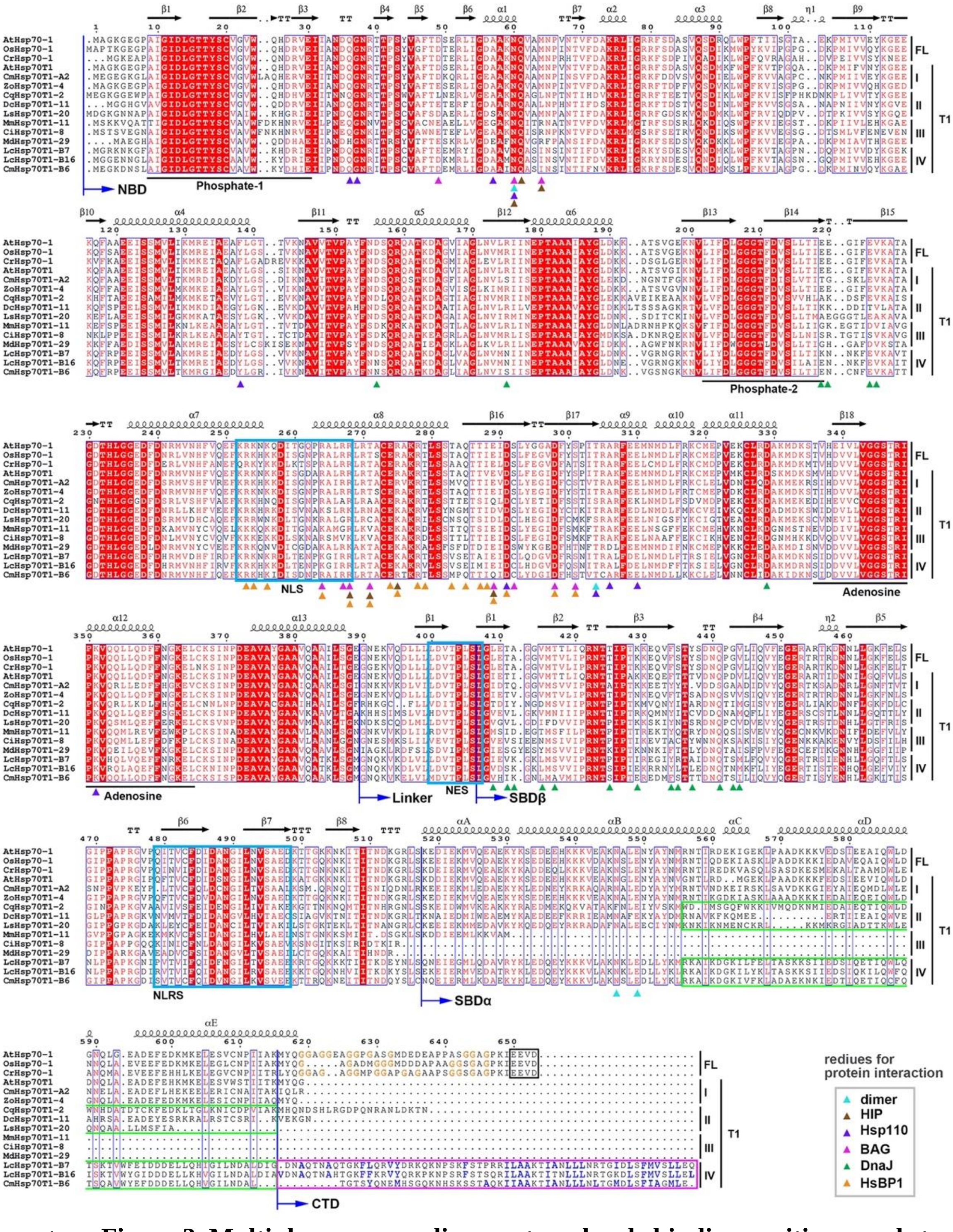

Supplementary Figure 3. Multiple sequence alignment, molecule binding positions, and structural characteristics of full-length cyHSP70s and the T1 variant of truncated cyHsp70s. The sequences employed for alignment correspond to those presented in Figure 2. The secondary structure positions, as predicted in the 3D structure of AtHsp70-1 (refer to Supplementary Figure 2), are indicated. Nucleotide-interacting positions within the Nucleotide-Binding Domain (NBD) are labeled as Phosphate-1, Phosphate-2, and Adenosine, corresponding to α-phosphate, β-phosphate, and adenosine binding sites, respectively. The regions analogous to SBDα (SBDαL) and the C-terminal extension are highlighted by unfilled green and magenta boxes, respectively.

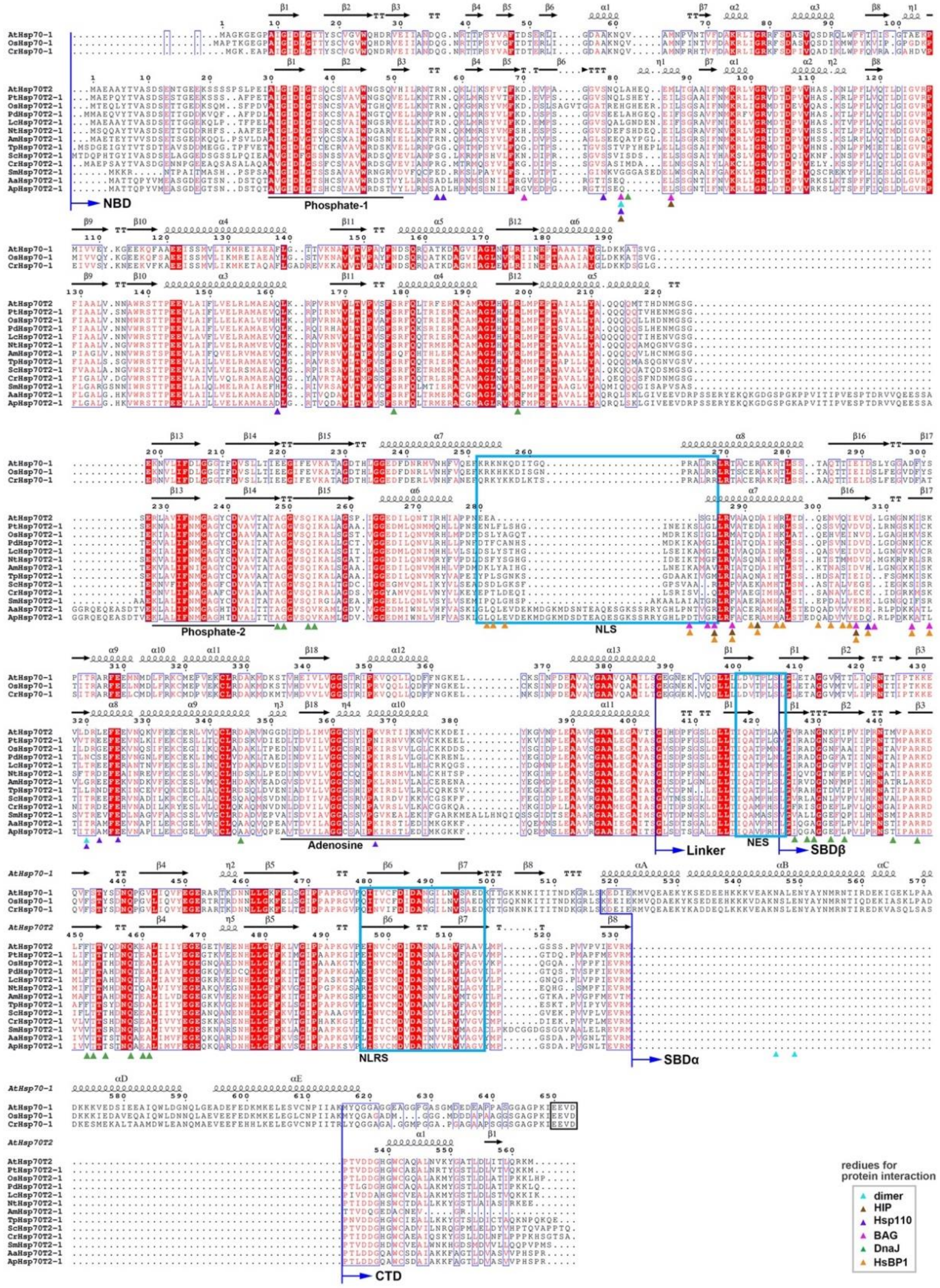

Supplementary Figure 4. Multiple sequence alignment, molecule binding positions, and structural characteristics of full-length cyHSP70s and the T2 variant of truncated cyHsp70s. The sequences analyzed were collected from *A. thaliana*, *Oryza sativa*, *Populus trichocarpa*, *Nicotiana tabacum*, *Phoenix dactylifera*, *Litsea cubeba*, *Amborella trichopoda*, *Thuja plicata*, *Selaginella moellendorffii*, *Salvinia cucullata*, *Anthoceros agrestis*, *Anthoceros punctatus* and *Chlamydomonas reinhardtii*. The secondary structure positions, as predicted in the 3D structure of AtHsp70-1 and AtHsp70T2 (refer to Supplementary Figure 2), are indicated. Nucleotide-interacting positions within the Nucleotide-Binding Domain (NBD) are labeled as Phosphate-1, Phosphate-2, and Adenosine, corresponding to α-phosphate, β-phosphate, and adenosine binding sites, respectively.

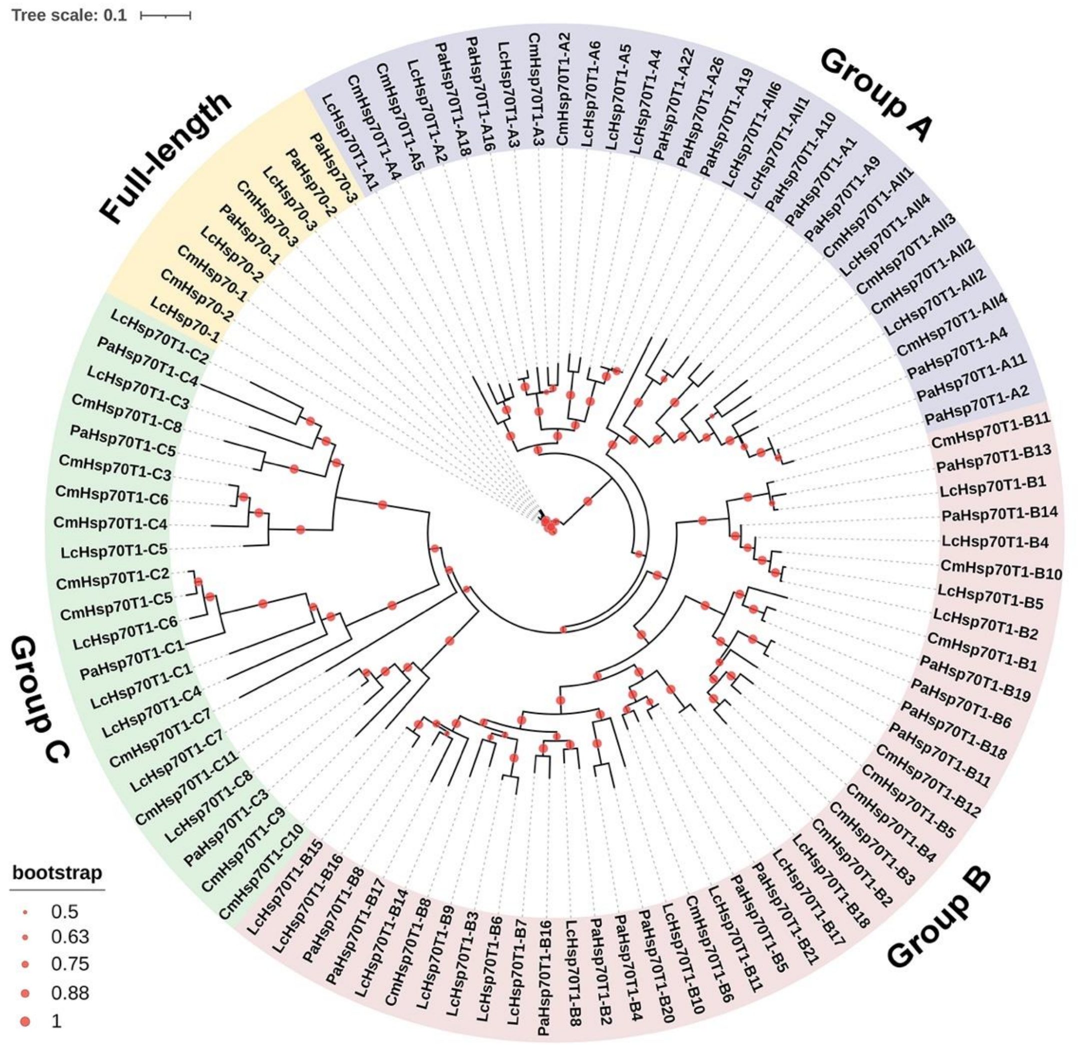

Supplementary Figure 5. Circular phylogenetic tree of cyHsp70T1 sequences in investigated Lauraceae species. Protein sequences of full-length cyHsp70 and cyHsp70T1 obtained from three Lauraceae species (*Litsea cubeba*, *Cinnamomum micranthum*, and *Persea americana*) were aligned using the MAFFT program. The Neighbor-Joining (NJ) method was employed to generate phylogenetic tree, with support from 1,000 bootstrap resampling. Branches with bootstrap values exceeding 0.5 are indicated by red dots. The sequences of full-length cyHsp70 are labeled as full-length. The scale bar represents 0.1 amino acid substitutions per site.

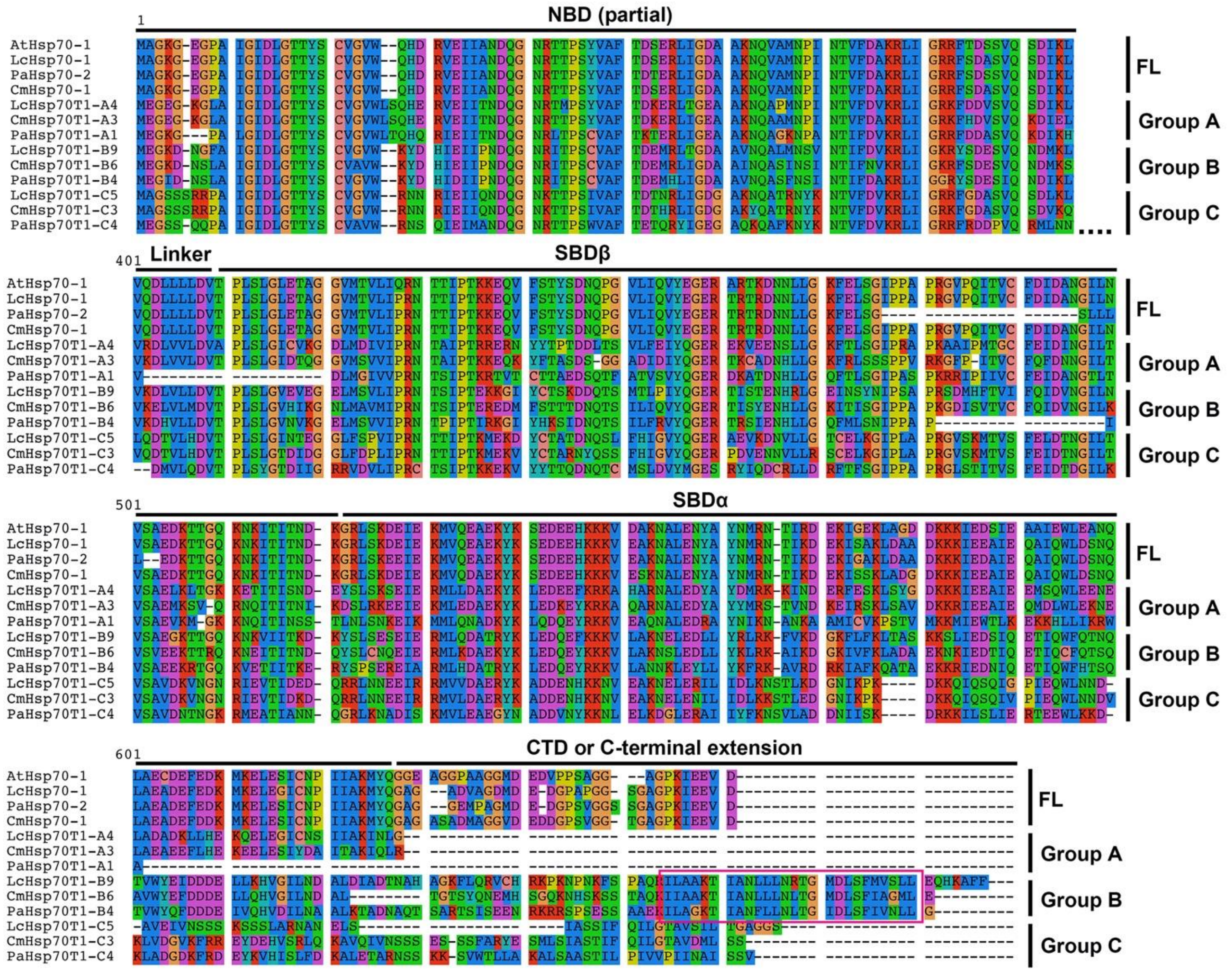

Supplementary Figure 6. Multiple sequence alignment of full-length cyHsp70 and cyHsp70T1 protein sequences in Lauraceae species. Protein sequences of cyHsp70T1 from three Lauraceae groups (Group A, B, and C) sourced from *Litsea cubeba*, *Cinnamomum micranthum*, and *Persea americana* were aligned using the MUSCLE program. The alignment results were visualized using the Seaview program. The conserved leucine-rich region of the Group B members is highlighted by an unfilled red box. Full-length cyHsp70s are indicated by FL.
